# Association of Tension-Adjusted Myocardial Work and Its Temporal Indices with LGE-Defined Myocardial Injury in Acute Myocarditis: A Proof-of-Concept Study

**DOI:** 10.3390/jcm15197455

**Published:** 2026-09-25

**Authors:** Kanza Awais, Konstantin Tripunovski, Tadeja Poropat Flerin, Andreja Černe Čercek, Borut Kirn

**Affiliations:** 1 Institute of Physiology, Faculty of Medicine, University of Ljubljana, 1000 Ljubljana, Slovenia; 2 Faculty of Medicine, University of Ljubljana, 1000 Ljubljana, Slovenia; konstantin.tripunovski@gmail.com; 3 Department of Radiology, University Medical Centre Ljubljana, 1000 Ljubljana, Slovenia; 4 Department of Cardiology, University Medical Centre Ljubljana, 1000 Ljubljana, Slovenia

**Keywords:** myocardial work index, ventricular geometry, wall stress, tension-adjusted myocardial work, stress–strain, myocardial injury, LGE

## Abstract

**Background:** Cardiac magnetic resonance (CMR) late gadolinium enhancement (LGE) is an important marker of myocardial injury in acute myocarditis (AM). Although echocardiographic myocardial work (MWI) provides a non-invasive assessment of left ventricular (LV) mechanics, conventional MWI does not account for ventricular geometry or temporal distribution of myocardial work. These factors may provide additional physiological information relevant to myocardial injury. **Objective:** To investigate the association of conventional and geometry- and timing-informed myocardial work measures with LGE-defined injury in AM in a preliminary proof-of-concept study. **Methods:** In this retrospective, single-center study, 41 patients with AM underwent echocardiography and CMR within ±10 days. Conventional segmental MWI was derived from pressure-strain loops. Tension-adjusted myocardial work (TAMW) incorporated ventricular geometry using CMR-derived ventricular volume and wall volume into the myocardial work estimation. Front Loading Index (FLI) and Back Loading Index (BLI) quantified the relative contribution of constructive work during the initial and final 20% of the ejection phase, respectively. Associations between myocardial work indicators and segmental LGE were assessed using Spearman correlation and linear mixed-effects (LME) models with patient-specific random intercepts. Multiple comparisons were addressed using Holm correction. Additional analyses examined segmental localization, within- and between-patient variability, patient-specific slopes and the influence of extreme observations. **Results:** Spearman correlations between LGE and MWI (ρ = −0.09, *p* = 0.01) and TAMW (ρ = −0.06, *p* = 0.08) were weak. In LME analysis, greater LGE was associated with lower MWI (β = −2.57, 95% CI −4.71 to −0.42, Holm-adjusted *p* = 0.038) and lower TAMW (β = −11.09, 95% CI −21.24 to −0.94, Holm-adjusted *p* = 0.038). Exploratory model comparison using LGE as the common outcome showed comparable model support for standardized MWI and TAMW (AIC = 6264.7 and 6265.7 respectively; ΔAIC = 0.97). FLI was not significantly associated with LGE in the LME analysis (β = −0.00021, 95% CI: −0.000558 to 0.000147, Holm-adjusted *p* = 0.253), whereas BLI showed a significant positive association with LGE (β = 0.000127, 95% CI: 0.000065 to 0.000190, Holm-adjusted *p* < 0.001) The BLI association was not maintained after accounting for segmental localization. **Conclusions:** In this preliminary proof-of-concept, MWI and TAMW showed weak inverse associations with segmental LGE in the primary analysis, while BLI demonstrated an exploratory positive association with LGE and FLI was not independently associated with LGE. These findings demonstrate the feasibility of incorporating ventricular geometry and temporal distribution into myocardial work assessment but do not establish superiority or incremental value over conventional MWI. Further prospective studies are required to determine the reproducibility, physiological significance, and clinical utility of these additional dimensions.

## 1. Introduction

Acute myocarditis (AM) is characterized by heterogenous myocardial injury, with regional involvement that may include inflammation, edema, necrosis and fibrosis [1,2,3,4]. These tissue abnormalities can affect ventricular structure and regional mechanics, sometimes without marked impairment of conventional measures of left ventricular (LV) function [5]. This highlights the need for sensitive approaches capable of detecting regional mechanical consequences of myocardial injury [3].

Cardiac magnetic resonance (CMR), particularly late gadolinium enhancement (LGE), provides an established approach for characterizing focal myocardial injury and its distribution [6]. CMR also permits the assessment of ventricular volumes and geometry. However, its cost, availability and limited suitability for repeated examinations support the investigation of complementary echocardiographic measures that can characterize myocardial mechanics. Conventional echocardiographic parameters such as ejection fraction and global longitudinal strain may not fully capture regional mechanical alterations [7,8]. Myocardial work index (MWI), derived from pressure-strain loop (PSL) analysis, integrates myocardial deformation with estimated LV pressure and therefore provides information beyond conventional strain measures [9]. Nevertheless, conventional MWI primarily quantifies the magnitude of myocardial work generated and does not explicitly incorporate other factors that may influence myocardial loading [10] or the timing of work generation.

Ventricular geometry is one such factor. According to the Law of Laplace, myocardial wall stress depends on ventricular pressure, ventricular dimension and wall thickness [10,11]. Consequently, similar pressure-strain behavior may occur under different mechanical loading conditions. We previously introduced the tension-adjusted myocardial work (TAMW) index, which incorporates ventricular geometry and strain-dependent scaling into myocardial work estimation [12]. A second potentially relevant dimension is the temporal distribution of constructive work. Regional myocardial injury may alter the coordination and timing of contraction, potentially redistributing constructive work across the ejection phase. Quantifying this distribution may therefore provide information that is not captured by the total magnitude of myocardial work.

Accordingly, this study extends conventional myocardial work assessment into a physiological myocardial work framework as shown in Figure 1, comprising TAMW as a geometry-related measure and two temporal indices, the Front Loading Index (FLI) and Back Loading Index (BLI), which quantify the relative contribution of constructive work during the early and late portions of ejection, respectively. We evaluated the associations between these indicators and segmental LGE-defined myocardial injury and compared the geometry-informed TAMW with conventional MWI. We hypothesized that incorporating ventricular geometry and the temporal distribution of constructive work could provide complementary information on the myocardial mechanical alterations associated with myocardial injury.

## 2. Methods

We retrospectively analyzed 50 cases of AM treated between January 2017 and January 2024. Patients were excluded because of incomplete or poor-quality echocardiographic recordings (*n* = 4), absence of cine short-axis CMR images (*n* = 2), or an interval outside ±10 days between echocardiography and CMR (*n* = 3), resulting in a final cohort of 41 cases.

All patients underwent clinical assessment, laboratory testing, electrocardiography (ECG), transthoracic echocardiography, and cardiac magnetic resonance (CMR) imaging as part of their diagnostic evaluation at the time of admission. Echocardiography and CMR examinations were performed within ±10 days of each other in the included patients. AM was confirmed by an experienced cardiologist based on the clinical presentation and a combination of biochemical, ECG and CMR findings. The study inclusion criteria were elevated troponin I levels, ECG changes suggestive of AM and CMR findings consistent with AM according to the Lake Louise Criteria [13]. Patients with documented coronary artery disease were excluded. During clinical management, patients received standard medical treatment according to clinical indication, which included angiotensin-converting enzyme (ACE) inhibitors and beta-blockers to optimize cardiac remodeling and reduce the risk of cardiac arrhythmia where appropriate. Some patients additionally received immunomodulatory treatment with intravenous immunoglobulins (IVIG). This study was conducted in accordance with the institutional ethics committee requirements for retrospective analysis of anonymized clinical images. Ethical approval was obtained from the local ethics committee (Resolution No. 0120-341/2024-2711-5, approved on 3 September 2024).

### 2.1. Transthoracic Echocardiography

Transthoracic echocardiographic examinations were performed using a GE vivid E95 system (GE Healthcare, Horten, Norway) equipped with a 4Vc phased-array transducer. Standard apical 2-, 3-, and 4-chamber views were acquired to assess the LV function and myocardial deformation.

Strain was calculated using EchoPac software (Version 206; GE Healthcare, Horten, Norway) with the automatic function imaging (AFI) protocol. Myocardial borders were initially traced automatically and subsequently reviewed and manually corrected when required (Figure 2a–c). Strain was referenced to the end-diastole, determined using the AVO event and ECG R wave. Segmental longitudinal strain patterns with peak strain values were acquired for all 18 LV segments according to the standard 18-segment model, with GLS calculated as an average of the segmental values (Figure 2d–f).

### 2.2. Conventional Myocardial Work Assessment

Brachial blood pressure (BP) was measured before echocardiographic acquisition according to the standard protocol [14]. LV pressure was estimated non-invasively from BP values and valvular event timings obtained from the apical 3-chamber view, following established pressure-strain loop methodology [9]. Pressure-strain loops (PS loops) were generated by combining the estimated LV pressure curves with the segmental strain [9], as shown in Figure 3a. Conventional MWI was calculated as the integrated area of pressure-strain loop over the systolic period from mitral valve closure (MVC) to mitral valve opening (MVO). Segmental MWI values were calculated separately for each of the 18 LV segments (Figure 3b). Global MWI was obtained by averaging the corresponding segmental values.

### 2.3. Cardiac Magnetic Resonance Imaging

CMR examination was performed using a 3 Tesla (T) Siemens Healthineers TrioTim scanner. ECG-gated steady-state free precession cine images were acquired in standard short-axis orientation covering the entire LV. Following intravenous administration of 0.1 mmol/kg gadolinium-based contrast agent (Gadovist), LGE images were acquired using phase-sensitive inversion recovery (PSIR) sequences.

LGE images were analyzed using Qmass software (Version no. 2025.1.14.6; Medis Medical Imaging Systems, Leiden, The Netherlands) as demonstrated in Figure 4. Endocardial and epicardial contours were delineated on cine images at the end-diastole and end-systole and manually corrected when necessary (Figure 4a,c). LGE extent was quantified using a threshold of 3 standard deviations (SD) relative to visually normal remote myocardium (Figure 4a). LGE was quantified for each of the 18 LV segments and expressed as the percentage of myocardial tissue demonstrating LGE within each segment (Figure 4b). The mean of the 18 segmental values was used to obtain global LGE extent.

Left ventricular end-diastolic volume (EDV), end-systolic volume (ESV), stroke volume, ejection fraction, and LV mass were also obtained from cine images (Figure 4c). Myocardial wall volume ($V_{w})$ was estimated from LV mass using a myocardial specific gravity of 1.05 g/mL. All these measurements were obtained according to the standard recommendations for CMR image analysis [15].

All echocardiographic, CMR, and derived volumetric parameters were subsequently transferred to MATLAB R2024a (Version no. 24.1) for the offline post-processing analysis.

### 2.4. Proposed Physiological Myocardial Work Framework

To extend conventional myocardial work analysis, we developed a physiological myocardial work approach that incorporates additional determinants influencing myocardial work generation. This approach consists of two complementary components: (1) tension-adjusted myocardial work (TAMW), which accounts for ventricular geometry in the myocardial work estimation, and (2) temporal indices (FLI, BLI), which characterize the distribution of constructive work during the ejection phase.

### 2.5. Tension-Adjusted Myocardial Work

The TAMW index was calculated by extending the conventional pressure-strain approach to incorporate ventricular geometry using a Law-of-Laplace-based one-fiber model as described in our previous study [12] (Figure 5a):

$$TAMW\;\left(t\right)=-\lambda \int_{0}^{t}P\left(t^{\prime }\right){\left(1+\varepsilon \left(t^{\prime }\right)\right)}^{2}\left(\frac{d\varepsilon }{dt^{\prime }}\right)dt^{\prime }\;$$

where *P* is estimated LV pressure, ε is longitudinal strain, and λ is the geometric scaling factor defined as

$$\lambda =1+3\frac{EDV}{V_{w}}$$

where EDV represents end diastolic volume and *V_w_* is the myocardial wall volume. The term (1+ ε)^2^ accounts for deformation-dependent changes in myocardial geometry during contraction.

TAMW was integrated over the systolic interval from MVC to MVO. Segmental TAMW values were computed for all 18 myocardial segments (Figure 5b) and averaged to acquire global TAMW. Stress–strain loop and representative segmental TAMW values are illustrated in an 18-segment bullseye plot as shown in Figure 5a and Figure 5b, respectively.

### 2.6. Temporal Distribution of Constructive Myocardial Work

For temporal characterization of constructive myocardial work, two novel exploratory indices were derived from the instantaneous TAMW curve. The ejection phase was defined from aortic valve opening (AVO) to aortic valve closure (AVC), and only positive (constructive) instantaneous work values were included in the analysis, with negative values (representing wasted work) set to zero prior to integration.

To account for differences in heart rate and acquisition frame rate between subjects, segmental instantaneous TAMW curves during ejection were resampled onto a normalized 100-point time grid (0–100% of ejection duration) using linear interpolation. This normalization allowed consistent identification and comparison of the early and late ejection phases across all segments before calculation of temporal indices.

The initial and final 20% portions of the ejection phase were selected to characterize the temporal distribution of constructive work during distinct phases of ventricular contraction. The early ejection phase is associated with rapid pressure development and initial ventricular emptying, whereas the late ejection phase represents the terminal phase of systolic contraction preceding AVC. A 20% ejection phase window was chosen as a practical compromise between physiological specificity and measurement robustness, allowing characterization of regional shifts in work timing while minimizing sensitivity to temporal noise, frame rate limitations, and uncertainty in valve event detection.

Front Loading Index (FLI) was defined as the ratio of constructive work accumulated during the initial 20% of the ejection phase to the total constructive work during ejection and is shown by the shaded blue area in Figure 6a.

Back Loading Index (BLI) was defined as the ratio of constructive work during the last 20% of the ejection window to total constructive work during ejection, illustrated as the shaded green area in Figure 6b.

All FLI and BLI values were calculated at the segmental level for each of the 18 LV myocardial segments.

Segmental conventional MWI and TAMW, together with the corresponding FLI and BLI indices, were correlated with segmental LGE extent.

### 2.7. Statistical Analysis

Statistical analyses were performed using MATLAB (R2024a Version no. 24.1; MathWorks, Natick, Massachusetts, USA). All primary analyses were conducted at the segmental level, using the 18 segmental LGE values and corresponding MWI, TAMW, FLI, and BLI measurements. Data distribution was assessed using the Lilliefors test. Continuous variables are presented as mean ± standard deviation. Because the variables were not normally distributed, Spearman’s rank correlation was used to assess unadjusted association between segmental LGE and each myocardial work derived indicator. To account for the clustering of multiple myocardial segments within individual patients, linear mixed effects (LME) models with patient-specific random intercepts were used [3]. Segmental LGE extent was included as a fixed effect, and the corresponding myocardial work indicator was included as the dependent variable:

*Y*_ij_ = (*β*_0_ + u_i_) + *β*_1_ (*LGE*_ij_) +ϵ_ij_

where Y*_ij_* represents the specific mechanical or temporal index for segment *j* in patient *i,* u*_i_* is the patient-specific random intercept accounting for correlation among segments within the same patient, and *β*_1_ represents the estimated change in the corresponding myocardial work-derived index per one percentage-point increase in segmental LGE. Effect estimates, 95% confidence interval (CI) and *p* values were acquired.

For MWI and TAMW, extreme observations were identified separately for each measure using a threshold of ±3 SD from its respective group mean. The overlapping exclusions resulted in five unique segments being excluded from the 738 available segments, leaving 733 segments for the primary MWI and TAMW analyses. For FLI and BLI, the same prespecified ±3 SD criterion was applied independently to each temporal index. This resulted in 728 segments for FLI and 726 segments for BLI, with all 41 patients represented in both analyses.

To account for multiple comparisons, Holm adjustment was applied to the *p* values from the two primary LME analyses of MWI and TAMW. Holm adjustment was applied separately to the FLI and BLI analyses.

For MWI and TAMW, an exploratory model comparison was additionally performed with LGE as the common dependent variable. Standardized MWI and TAMW were entered separately as predictors in LME models using the same observations and a patient-level random-effects structure. Akaike Information Criterion (AIC) was used to compare model support, with lower AIC indicating better relative model support. This analysis was used only as an exploratory comparison and not to establish superiority of TAMW over conventional MWI.

For exploratory categorical analyses, myocardial segments were stratified according to LGE extent using a 20% threshold (≤20% vs. >20%). This threshold was used as an exploratory cut-off to distinguish relatively lower from higher LGE burden and to facilitate visualization of differences in temporal work indices. Continuous LGE analyses were considered primary, while categorical analyses were considered supportive exploratory analyses. Group differences were evaluated using LME models with patient-specific random intercepts.

Additional analyses addressing segmental and patient-level variability examined the influence of segment localization, within- and between-patient LGE variation, and patient-specific slopes. Segment localization was incorporated as a categorical fixed effect in the LME models. To distinguish regional within-patient associations from differences in overall myocardial injury between patients, segmental LGE was decomposed into the patient-level mean component and the segment-specific deviation from that patient mean [16]. Both components were included simultaneously as fixed effects in separate LME models for MWI and TAMW, with patient-specific random intercepts. Patient-specific random-slope models were additionally fitted to allow the LGE–myocardial work relationship to vary between patients. Analyses including all available segments were also performed to assess the robustness of the primary MWI and TAMW findings.

All statistical tests were two-sided, and *p* < 0.05 was considered statistically significant.

## 3. Results

The study cohort consisted of 41 patients with AM. Echocardiography and CMR examination were performed within ±10 days for all patients. Segmental LGE extent and myocardial work-derived indicators were obtained for each patient.

Baseline characteristics and imaging parameters are represented in the Table 1.

### 3.1. Association Between Myocardial Work Framework and LGE-Defined Injury

Of the 738 initially available segments from 41 cases, 5 (0.68%) were excluded based on the ±3 SD criterion, leaving 733 segments for the primary MWI and TAMW analyses. 6 segments had no detectable LGE, and no observations were missing for LGE, MWI and TAMW. Simple segmental correlation analysis showed weak inverse relation between LGE extent and both conventional MWI (ρ = −0.09, *p* = 0.01) and TAMW (ρ = −0.06, *p* = 0.08).

In LME models accounting for clustering of segments within patients (Figure 7), greater LGE extent was associated with lower MWI (β = −2.57, 95% CI −4.71 to −0.42, *p* = 0.019) and lower TAMW (β = −11.09, 95% CI −21.24 to −0.94, *p* = 0.032). After Holm adjustment for the two primary comparisons, both associations remained statistically significant (adjusted *p* = 0.038 for each).

Additional analyses addressing segmental and patient-level variability showed that incorporating segment localization yielded consistent inverse associations for both MWI (β = −4.06, 95% CI −6.23 to −1.89, *p* < 0.001) and TAMW (β = −18.37, 95% CI −28.67 to −8.06, *p* < 0.001). To distinguish regional within-patient association from differences in overall myocardial injury between patients, segmental LGE was decomposed into patient-level mean LGE and the segment-specific deviation from that patient mean. In mixed-effects models including both components and a patient-level random intercept, the within-patient component of LGE remained inversely associated with MWI (β = −2.53, 95% CI: −4.73 to −0.32, *p* = 0.025) and TAMW (β = −11.40, 95% CI: −21.81 to −0.99, *p* = 0.032). In contrast, the between-patient component was not significantly associated with either MWI (β = −3.29, 95% CI: −12.71 to 6.13, *p* = 0.493) or TAMW (β = −5.03, 95% CI: −51.16 to 41.11, *p* = 0.831).

Random-slope models demonstrated significant between-patient variability in the LGE–myocardial work relationship for both measures, with likelihood-ratio comparisons favoring models that allowed patient-specific slopes.

In a sensitivity analysis including all 738 available segments, the direction of both associations remained unchanged, although the effect estimates were attenuated and neither association remained statistically significant (MWI: β = −1.89, 95% CI: −4.07 to 0.29, *p* = 0.089; TAMW: β = −7.67, 95% CI: −18.05 to 2.71, *p* = 0.147).

Finally, an exploratory model comparison using LGE as the common dependent variable showed similar model support for standardized MWI and TAMW (AIC = 6264.7 and 6265.7, respectively; ΔAIC = 0.97), providing no evidence of a meaningful difference in model support.

### 3.2. Temporal Distribution of Constructive Myocardial Work and LGE Defined Injury

The temporal distribution of constructive myocardial work was evaluated at the segmental level using FLI and BLI (Figure 8). No observations were missing for LGE, FLI or BLI. After ±3 SD outlier exclusion, 10 segments were excluded from the FLI analysis, leaving 728 segments from 41 patients, and 12 segments were excluded from the BLI analysis, leaving 726 segments from 41 patients for BLI analysis. FLI showed a weak inverse Spearman correlation with LGE extent (ρ = −0.07, *p* = 0.047). In contrast, BLI showed a positive correlation (ρ = 0.20, *p* < 0.001).

In LME analysis, FLI was not significantly associated with LGE (β = −0.00021, 95% CI: −0.000558 to 0.000147, *p* = 0.253) (Figure 8a), whereas a significant positive association was found between BLI and LGE (β = 0.000127, 95% CI: 0.000065 to 0.000190, *p* < 0.001), indicating that greater LGE extent was associated with an increased relative contribution of constructive work during the final 20% of the ejection phase (Figure 8b). After Holm adjustment, FLI remained non-significant (adjusted *p* = 0.253), while the association between BLI and LGE remained significant (adjusted *p* = 0.000128).

When segmental localization was additionally incorporated, neither FLI (β = 0.00690, 95% CI: −0.02699 to 0.04080, *p* = 0.689) nor BLI (β = −0.00360, 95% CI: −0.01110 to 0.00390, *p* = 0.346) remained significantly associated with LGE. Patient-specific random-slope models indicated significant between-patient variability in the LGE-temporal index relationships for both FLI (likelihood-ratio test: X^2^ = 7.85, df = 2, *p*—0.0198) and BLI (X^2^ = 13.05, df = 2, *p* = 0.00147). In these models, FLI remained non-significant (β = −0.000117, 95% CI: −0.000545 to 0.000311, *p* = 0.592), whereas BLI remained positively associated with LGE (β = 0.000114, 95% CI: 0.000035 to 0.000192, *p* = 0.0046).

To further illustrate differences according to LGE burden, myocardial segments were stratified using an exploratory 20% threshold as shown in Figure 9. FLI was modestly lower in segments with >20% LGE compared with those with ≤20% LGE, but this difference did not reach statistical significance (β = −0.0126, 95% CI: −0.0277 to 0.0024, Holm-adjusted *p* = 0.100). In contrast, BLI was higher in segments with >20% LGE (β = 0.00614, 95% CI: 0.00348 to 0.00880, Holm-adjusted *p* = 1.36 × 10^−5^).

## 4. Discussion

The primary findings of this proof-of-concept study are that greater segmental LGE extent was associated with lower conventional MWI and TAMW in the primary analyses after exclusion of extreme observations, whereas these associations were attenuated when all available segments were included. The exploratory comparisons using LGE as a common outcome showed similar model support for MWI and TAMW, with no meaningful difference in AIC. Among the temporal indices, BLI showed a positive association with LGE in the primary mixed effects analysis, whereas FLI was not significantly associated with LGE. However, the association between BLI and LGE was no longer significant after accounting for segment localization. Additional analyses further indicated that the inverse MWI-LGE and TAMW-LGE associations were primarily attributable to within-patient regional variation, while the strength of the relationships varied between patients. Collectively, these findings demonstrate the feasibility of extending conventional myocardial work analysis to incorporate geometric and temporal dimensions but do not establish superiority or incremental diagnostic value over conventional MWI.

### 4.1. Magnitude of Myocardial Work and Role of Geometry

The observed inverse association between myocardial work and LGE extent is consistent with previous studies [17,18,19] demonstrating impaired regional myocardial mechanics in areas of myocardial injury. In the present study, both conventional MWI and TAMW were inversely associated with segmental LGE in the primary mixed-effects analyses, suggesting that greater myocardial injury burden is accompanied by reduced regional myocardial work. However, these associations were attenuated when all available segments were included, indicating that the findings were sensitive to the exclusion of extreme observations.

Conventional pressure-strain analysis primarily integrates myocardial deformation with estimated LV pressure but does not account for the influence of ventricular geometry on myocardial work [11]. In contrast, TAMW incorporates a geometric scaling factor (*λ*) derived from the relationship between EDV and *Vw*, together with dynamic (1+ ε)^2^. According to the Law of Laplace, myocardial wall stress depends not only on ventricular pressure but also on the relationship between cavity volume and wall thickness, which changes throughout the cardiac cycle [11,20]. Consequently, myocardial segments experiencing similar pressure-strain relationships may be subjected to different mechanical loading conditions. These geometric considerations may be relevant in acute myocarditis, where regional myocardial injury and remodeling can alter ventricular structure and mechanical conditions [21].

In the present study, however, the exploratory AIC comparison using LGE as the common dependent variable showed similar model support for conventional MWI and TAMW (ΔAIC = 0.97). Thus, the incorporation of ventricular geometry did not demonstrate superior model performance relative to conventional MWI in this cohort. Rather, these findings demonstrate the feasibility of incorporating geometric information into the myocardial work estimation and provide preliminary evidence that geometry-informed TAMW is associated with LGE-defined injury. Whether this additional physiological dimension provides clinically meaningful information beyond conventional MWI requires validation in larger and independent cohorts.

### 4.2. Temporal Redistribution of Myocardial Work

Beyond the magnitude and geometric dimensions of myocardial work, the present study explored whether LGE extent was associated with temporal distribution of constructive work during ejection. Conventional myocardial work parameters summarize constructive work over the systolic period [22] but do not describe how this constructive work is distributed throughout the systole. FLI and BLI were therefore examined as exploratory indices representing the relative contribution of constructive work during the early and late portions of the ejection phase, respectively.

FLI was not significantly associated with LGE extent in the primary mixed-effects analysis. Although a weak inverse relationship was observed in the unadjusted Spearman analysis, this association was not supported after accounting for the clustering of segments within patients. The exploratory analysis using the 20% LGE cut-off similarly showed a modest reduction in FLI in segments with >20% LGE, but this difference was not statistically significant. Thus, the present data do not provide evidence for a robust association between the relative contribution of early systolic constructive work and LGE extent.

In contrast, BLI demonstrated a significant positive association with LGE extent in the primary mixed-effects analysis, and segments with >20% LGE also demonstrated higher BLI values in the exploratory categorical analysis. This pattern suggests that greater LGE burden may be associated with a greater relative contribution of constructive work during the last portion of ejection. However, the BLI-LGE association was no longer significant after accounting for segment localization, indicating that the observed relationship is sensitive to anatomical location. Patient-specific random-slope analysis further indicated variability in the strength of the LGE–BLI relationship between patients. Therefore, the present findings should be regarded as exploratory evidence of a possible temporal alteration in myocardial work rather than evidence of a consistent or independent late-systolic shift.

The contrasting behavior of FLI and BLI nevertheless suggests that the temporal organization of constructive work may provide additional physiological dimensions for further investigation. Importantly, the present study does not establish that these temporal indices provide information beyond conventional MWI, and their independent physiological and clinical relevance remains to be determined.

Previous studies of ventricular dyssynchrony and cardiac resynchronization have demonstrated that the timing of regional contraction can influence the distribution of myocardial work without necessarily changing overall magnitude of myocardial shortening [23,24]. Although the underlying pathophysiology differs from AM, these observations provide a physiological rationale for examining the temporal work distribution in myocardial injury. Further studies are required to determine whether these temporal indices provide reproducible information beyond conventional measures of myocardial work and whether temporal work patterns change during myocardial recovery.

### 4.3. Potential Mechanisms Underlying Altered Temporal Work Distribution

The physiological basis of the observed difference between early and late ejection work distribution remains uncertain. One possible explanation is altered regional contraction and mechanical coordination. Previous myocardial work studies have demonstrated that the timing of myocardial shortening can influence regional work distribution, particularly when contraction occurs under heterogeneous loading conditions [25]. Regional myocardial injury could therefore alter the timing of contraction and the associated mechanical loading during the ejection phase.

Alternatively, redistribution of regional work could reflect an adaptive response to heterogenous mechanical loading, whereby realtively preserved myocardium may contribute differently to ventricular work to mainatin systolic ejection [26]. Conversely, altered regional coordination could produce mechanically inefficient or maladaptive contraction and consequently modify the temporal distribution of constructive work [27].

Importantly, the present study was not designed to distinguish between compensatory, maladaptive, or timing-related mechanisms. The observed increase in BLI should therefore not be interpreted as evidence of a specific physiological response. Further studies incorporating regional contraction timing, mechanical dyssynchrony, ventricular loading conditions and longitudinal changes in tissue injury will be required to determine the mechanisms underlying the observed temporal work pattern.

### 4.4. Interpretation of Additional Statistics

The additional analysis highlighted the importance of anatomical and patient-level variability when interpreting segmental relationships between myocardial injury and mechanics. Accounting for segment localization resulted in consistent inverse associations for both MWI and TAMW, suggesting that the observed relationships were not simply attributable to differences in anatomical location. This finding is consistent with our previous work showing that anatomical orientation mismatch and natural physiological heterogeneity can substantially influence the segmental relationship between CMR-derived tissue abnormalities and echocardiographic strain [3].

Decomposition of LGE into within- and between-patient components further showed that the inverse associations were primarily attributable to within-patient regional variation. Specifically, greater LGE relative to a patient’s own mean LGE was associated with lower MWI and TAMW, whereas the between-patient component was not significantly associated with either measure. This supports the interpretation that the observed associations were driven primarily by regional differences in myocardial injury and mechanics rather than differences in overall LGE burden between patients. Nevertheless, our previous study demonstrated substantial natural variability in segmental myocardial strain, with physiological variability exceeding the myocarditis-related change in strain, emphasizing the difficulty of distinguishing disease-related regional heterogeneities from background inter-individual variability [28].

At the same time, patient-specific random-slope analyses indicated that the strength of the LGE–myocardial work relationship varied between patients. Thus, although the within-patient associations were consistent at the population level, the magnitude of the relationship was not necessarily uniform across patients. This variability further emphasis the need for caution when interpreting a single segmental myocardial work value in isolation.

The sensitivity analysis including all available segments resulted in attenuation of the observed associations, and neither MWI nor TAMW remained statistically significant, although the direction of association remained consistent. This indicates that the primary findings were influenced to some extent by extreme observations excluded by the ±3 SD criterion. Together, these analyses highlight the influence of anatomical location, within- and between-patient variability, and extreme observations on segmental relationships between tissue injury and myocardial work. These sources of variability should therefore be considered when evaluating segmental myocardial work in relation to myocardial injury and support the need for validation in larger, independent cohorts.

### 4.5. Clinical Implications

Although CMR-LGE remains the reference standard for myocardial tissue assessment, its availability is limited by cost, accessibility, and the practicality of repeated examinations. In contrast, echocardiography is inexpensive, more widely available, and routinely used during the diagnosis and follow-up of patients with AM. The present study provides preliminary evidence that myocardial work analysis can be extended to incorporate geometric and temporal characteristics of ventricular mechanics. However, the study does not establish that these additional indices can replace CMR or provide incremental diagnostic value over conventional MWI.

The current implementation of TAMW requires CMR-derived geometric parameters and therefore does not yet represent a stand-alone echocardiographic measure. A potential pathway towards clinical translation would involve deriving the required geometric parameters from routine echocardiographic measurements. Echocardiographic estimation of LV EDV using the biplane Simpson method and LV mass from standard LV dimensions using the Devereux formula could potentially provide the inputs required to estimate wall volume and the geometric scaling factor used in TAMW. These echocardiography-derived measurements would first require validation against CMR-derived values, followed by assessment of the reproducibility and agreement of fully echocardiography-derived TAMW.

Subsequent prospective studies should evaluate TAMW and the temporal indices against multiparametric CMR tissue characterization and clinical outcomes. Such stepwise development could ultimately determine whether geometry- and timing-informed myocardial work measures provide reproducible physiological information and whether TAMW can ultimately be implemented using echocardiography-derived data alone for longitudinal assessment of patients with AM.

## 5. Limitation

This study has several limitations. First, it was a retrospective, single-center study with a relatively limited cohort, and the findings require confirmation in larger, independent populations. Prospective, multicenter studies are needed to validate the proposed myocardial work measures and to determine whether longitudinal changes in these measures reflect recovery or persistence of myocardial injury and provide clinically relevant information beyond conventional echocardiographic and CMR parameters.

Secondly, the analysis was performed at the segmental level, and the observed associations were influenced by segmental localization and patient-level variability. In addition, the primary MWI and TAMW associations were attenuated when all available segments were included, indicating sensitivity to the extreme observations excluded by the ± 3 SD criterion. Although the within-patient analyses supported regional associations between LGE and MWI/TAMW, patient-specific random-slope models indicated variability in the strength of these relationships between individuals.

Third, although the proposed physiological myocardial work framework incorporates ventricular geometry through a Laplace Law-based approach, it remains dependent on strain measurements derived from echocardiography and non-invasive estimation of the LV pressure. Consequently, the framework is still influenced by loading conditions and image quality, similar to the conventional pressure-strain-based myocardial work analysis. In addition, the ventricular geometric parameters required for TAMW, including EDV and *V_w,_* were obtained from CMR. Therefore, the current implementation cannot yet be considered a fully echocardiography-derived physiological myocardial work assessment. Future studies should investigate the feasibility of deriving these geometric parameters directly from echocardiography, enabling development of a completely echocardiography-derived implementation of TAMW.

Finally, the temporal component of the proposed framework represents an exploratory extension of myocardial work assessment and requires further validation. Although BLI demonstrated a significant association with LGE-defined myocardial injury in the primary mixed-effects analysis, this association was no longer significant after accounting for segmental localization, whereas FLI was not significantly associated with LGE in the primary mixed-effects analysis. The reproducibility, physiological basis, clinical applicability and prognostic value of FLI and BLI therefore remain to be established in larger, independent cohorts and across different myocardial diseases.

## 6. Conclusions

In this preliminary proof-of-concept study, conventional MWI and TAMW showed weak inverse associations with segmental LGE after exclusion of extreme observations, but these associations were attenuated when all available segments were included. BLI showed an exploratory positive association with LGE in the primary mixed-effects analysis, whereas FLI was not independently associated with LGE. The BLI association was not maintained after accounting for segmental localization. These findings demonstrate the feasibility of extending myocardial work analysis to incorporate ventricular geometry and temporal distribution of constructive work but do not establish added value or superiority over conventional MWI. Further prospective studies are required to determine the reproducibility, physiological significance, and clinical utility of these additional dimensions in AM.

## Figures and Tables

**Figure 1:**
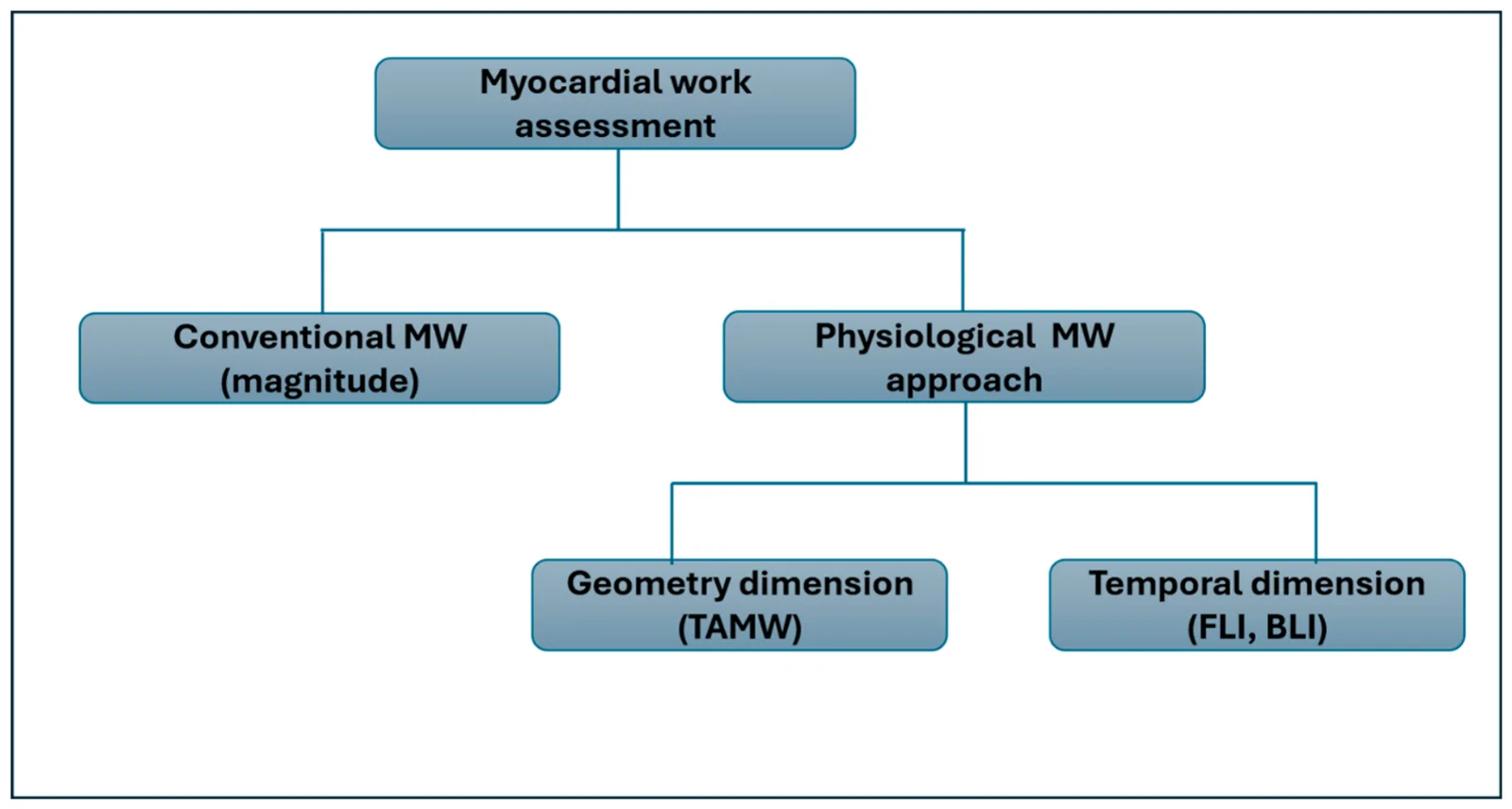
Conceptual overview of the multidimensional physiological myocardial work framework. Abbreviations: MW—myocardial work, TAMW—tension-adjusted myocardial work, FLI—Front Loading Index, BLI—Back Loading Index.

**Figure 2:**
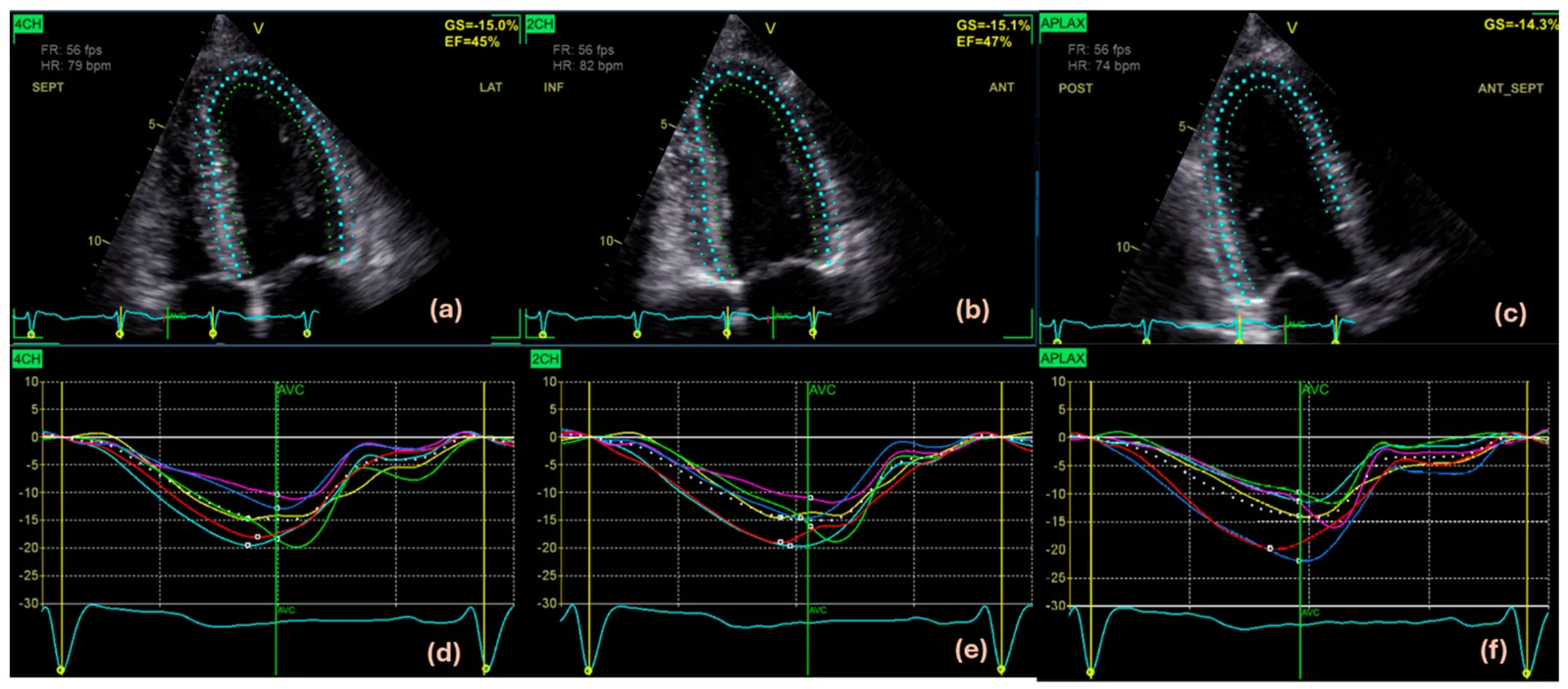
Strain analysis of Case 2 using EchoPac software (Version 206; GE Healthcare, Horten, Norway). Contours of LV were delineated in apical 4- (**a**), apical 2- (**b**), apical 3- (**c**) chamber views, along with six segmental strain traces from each view, respectively (**d**–**f**). Abbreviations: 4CH—apical 4 chamber, 2CH—apical 2 chamber, APLAX—apical long axis view (apical 3 chamber).

**Figure 3:**
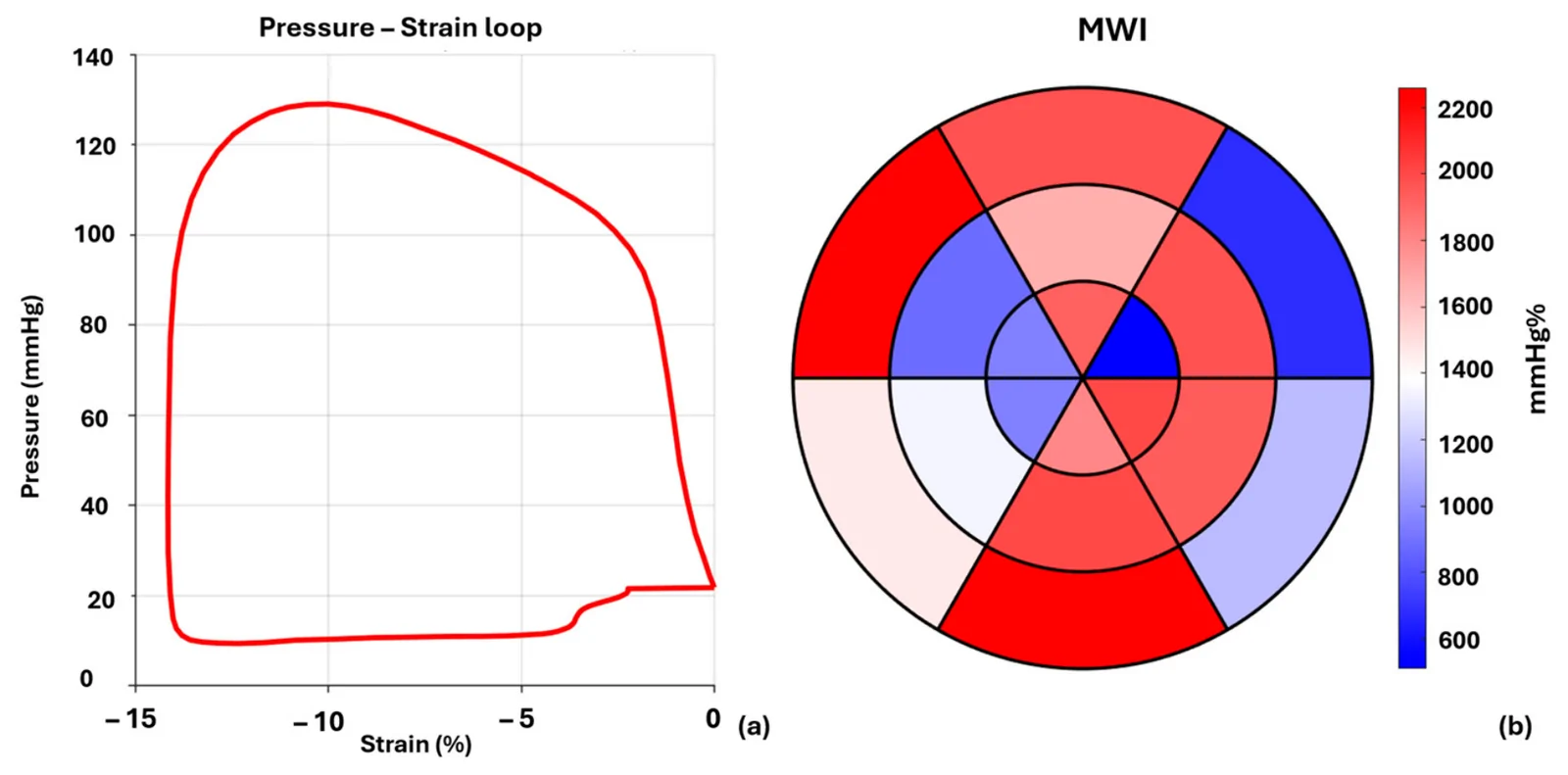
Pressure-strain loop of Case 2 (**a**) with 18-segment bullseye plot (**b**) where the inner ring represents apical segments, the mid ring represents mid regions, and the outer ring represents basal segments of the LV. Abbreviations: MWI—myocardial work index.

**Figure 4:**
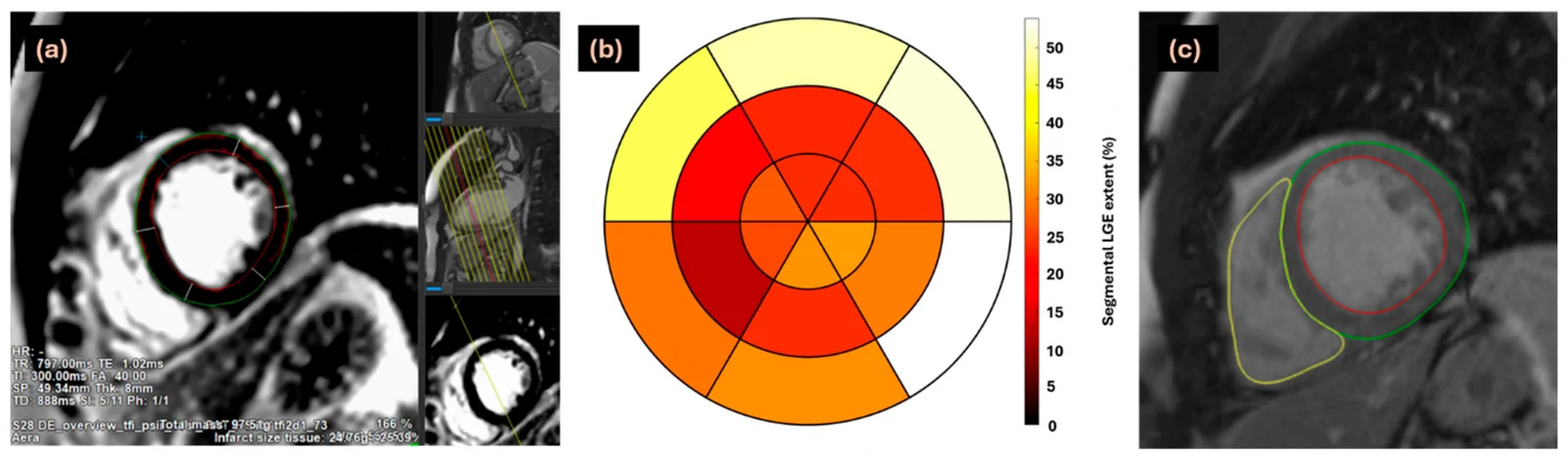
LGE quantification of LV in cine short axis view with epicardial and endocardial borders delineated in green and red, respectively (**a**). (**b**) Bullseye plot of segmental LGE extent values. (**c**) Delineated epicardial border (green), endocardial border (red) and right ventricle (yellow) to acquire volumetric parameters from Case 2. Abbreviation: LGE—late gadolinium enhancement.

**Figure 5:**
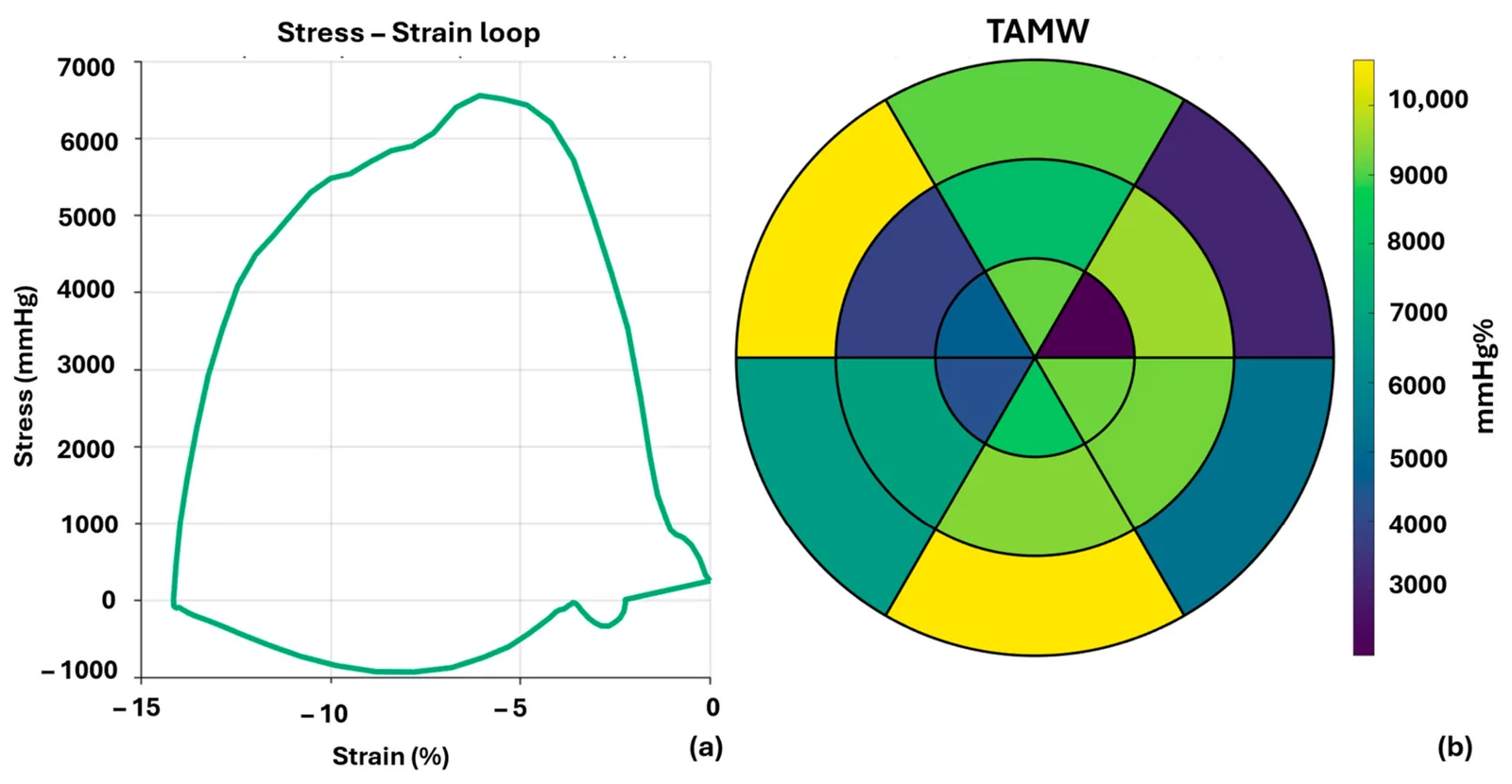
Stress–strain loop (**a**) along with segmental bullseye plot of TAMW values (**b**) of Case 2. Abbreviations: TAMW—tension-adjusted myocardial work.

**Figure 6:**
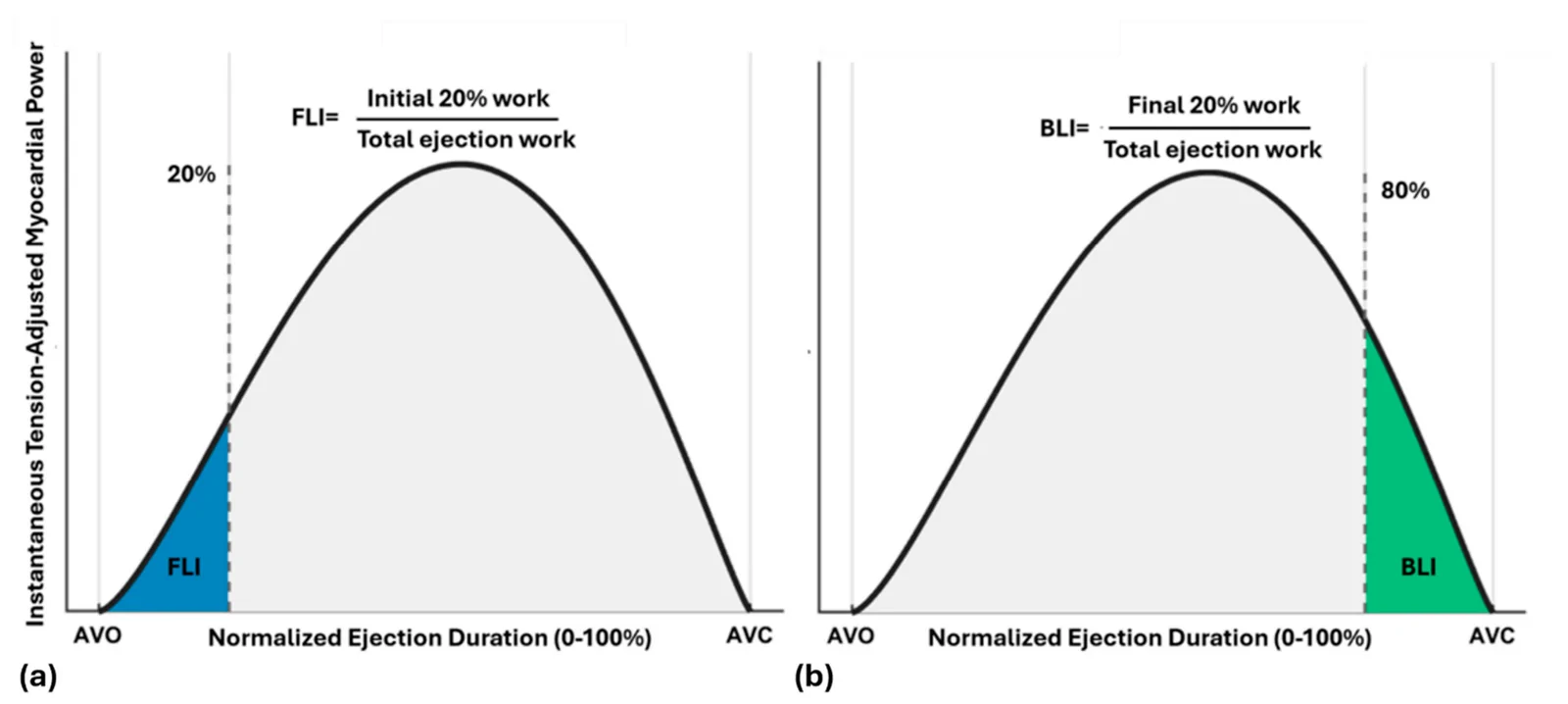
Methodological framework of quantifying temporal work indices FLI (**a**) and BLI (**b**). Abbreviations: FLI—Front Loading Index, BLI—Back Loading Index, AVO—aortic valve opening, AVC—aortic valve closure.

**Figure 7:**
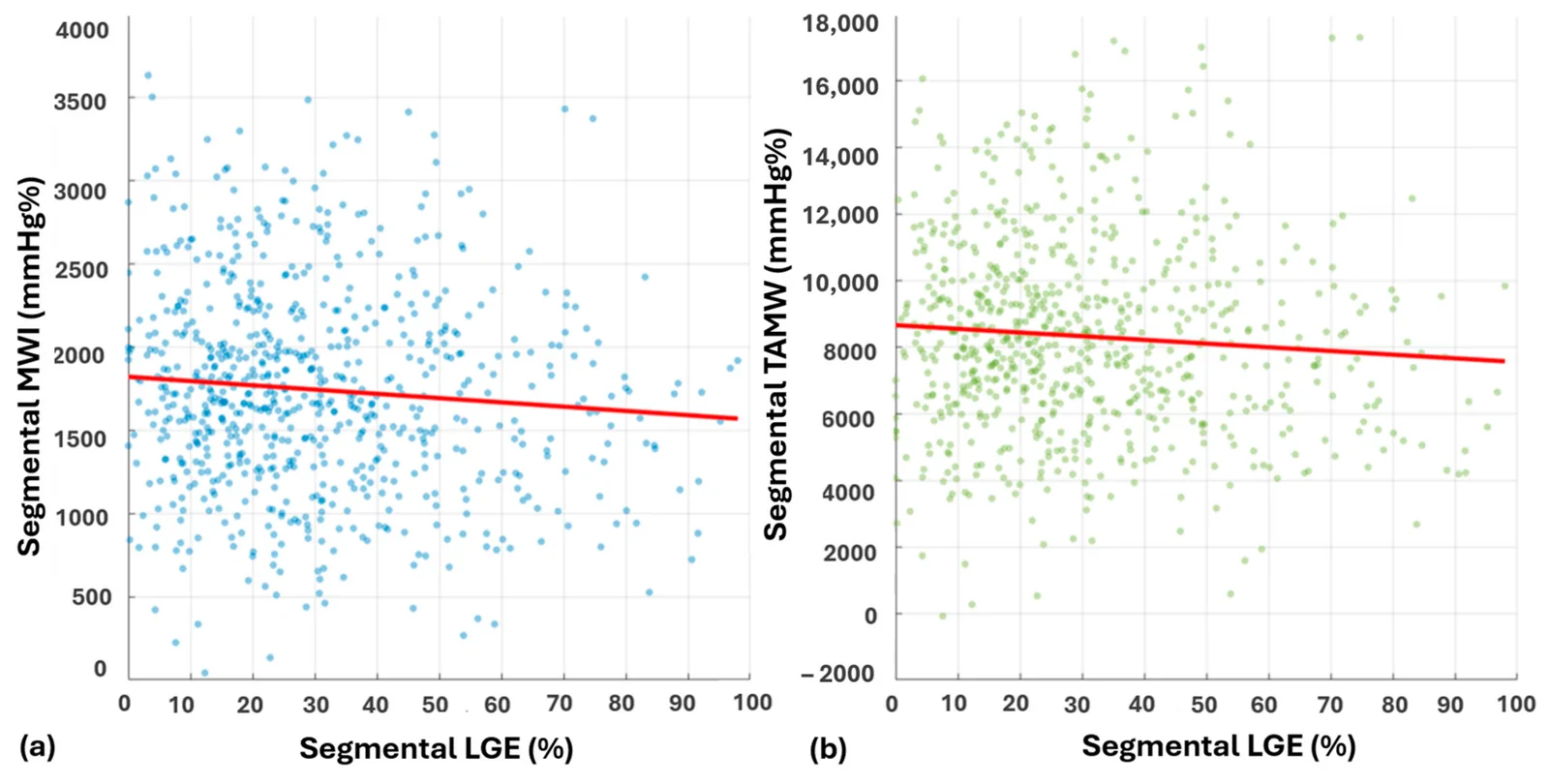
One-on-one segmental correlation of magnitude components of the proposed physiological myocardial work approach, i.e., MWI with LGE (**a**) and TAMW with LGE (**b**). Abbreviations: MWI—myocardial work index, TAMW—tension-adjusted myocardial work, LGE—late gadolinium enhancement.

**Figure 8:**
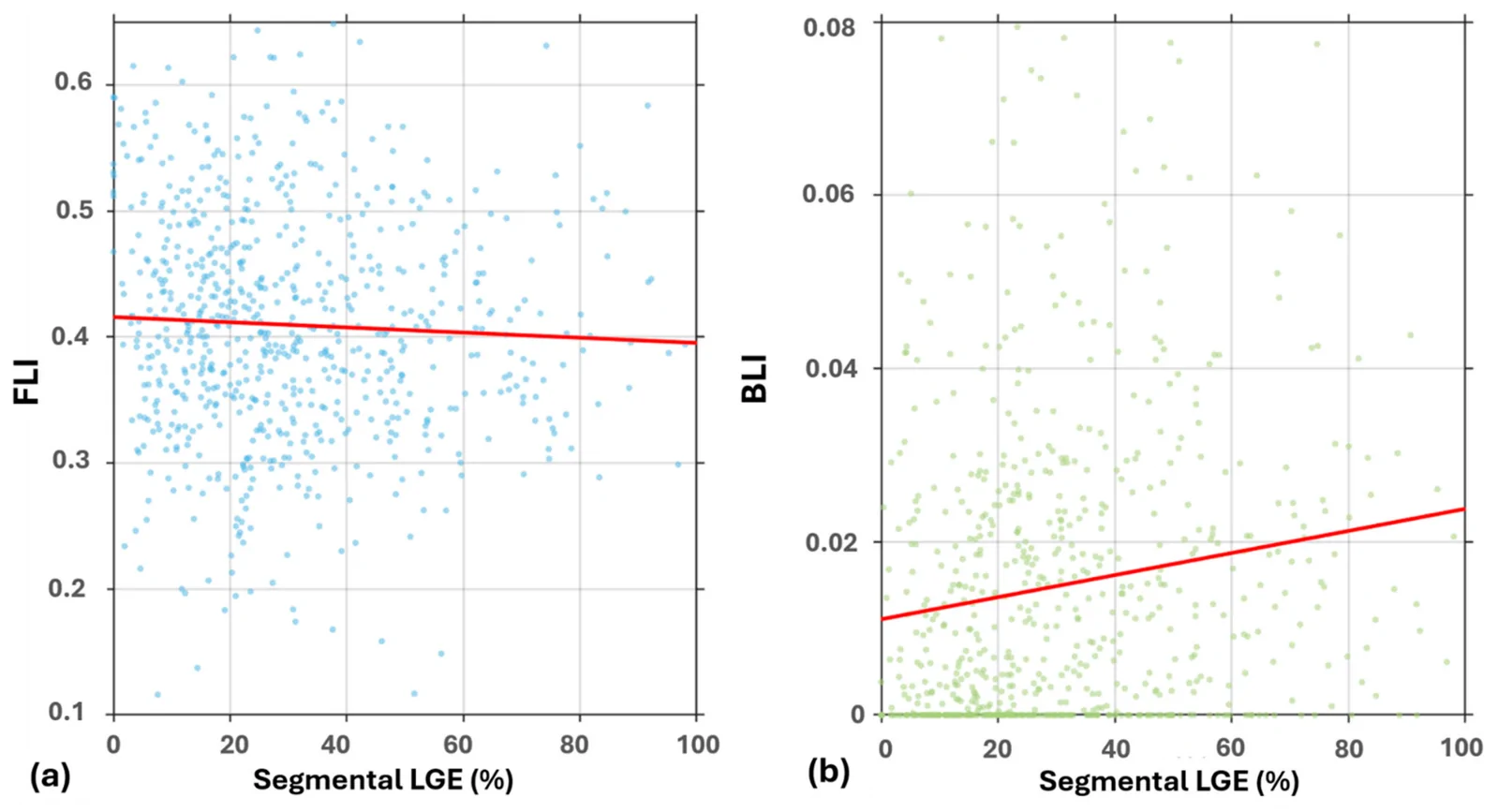
One-on-one segmental correlation of temporal myocardial work indices and LGE extent including FLI (**a**) and BLI (**b**). Abbreviations: FLI—Front Loading Index, BLI—Back Loading Index, LGE—late gadolinium enhancement.

**Figure 9:**
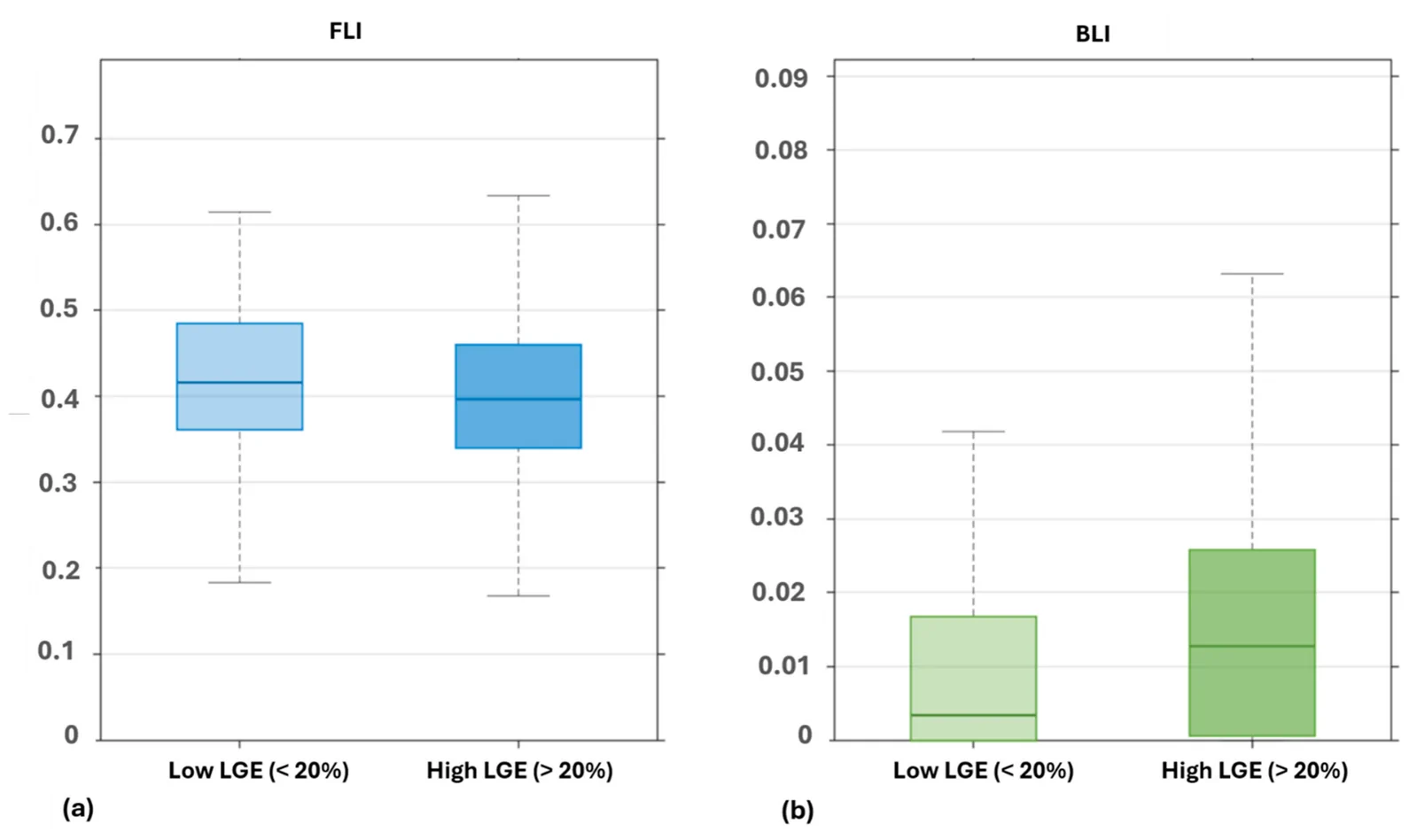
Side-by-side box plots comparing the proposed physiological work framework, i.e., FLI (**a**) and BLI (**b**), between myocardial segments with low (<20%) and high (>20%) LGE extent. Abbreviations: FLI—Front Loading Index, BLI—Back Loading Index, LGE—late gadolinium enhancement.

**Table 1:** Summary of patient characteristics. Abbreviations: BMI—body mass index, GLS—global longitudinal strain, EF—ejection fraction, NT-proBNP—N-terminal pro-B-type natriuretic peptide, CRP—C reactive protein, MWI—myocardial work index, TAMW—tension-adjusted myocardial work.

Parameters	Values
Gender (M/F)	36/5
Age	30.3 ±7.9
BMI (kg/m^2^)	26.7 ± 4.6
GLS (%)	−18.19 ± 2.32
EF (%)	57 ±8.7
Peak troponin (ng/mL)	16.8 ± 25.2
NT-proBNP (ng/L)	440 ± 560.9
Elevated CRP (mg/L)	71.5 ± 66.6
Creatinine (µmol/L)	80.9 ± 13
Global MWI (mmHg%)	1753.6 ± 423
Global TAMW (mmHg%)	8373.9 ± 2092.7
Global LGE extent (%)	29.4 ± 13.7

## Data Availability

The original contributions presented in this study are included in the article. Further inquiries can be directed to the corresponding author.
